# Repeatability and measurement error in the assessment of choline and betaine dietary intake: the Atherosclerosis Risk in Communities (ARIC) Study

**DOI:** 10.1186/1475-2891-8-14

**Published:** 2009-02-20

**Authors:** Aurelian Bidulescu, Lloyd E Chambless, Anna Maria Siega-Riz, Steven H Zeisel, Gerardo Heiss

**Affiliations:** 1 Cardiovascular Research Institute and Department of Community Health and Preventive Medicine, Morehouse School of Medicine, Atlanta, GA, USA; 2 Department of Biostatistics, School of Public Health, University of North Carolina, Chapel Hill, NC, USA; 3 Department of Epidemiology, School of Public Health, University of North Carolina at Chapel Hill, NC, USA; 4 Department of Nutrition, School of Public Health, University of North Carolina, Chapel Hill, NC, USA

## Abstract

**Background:**

The repeatability of a risk factor measurement affects the ability to accurately ascertain its association with a specific outcome. Choline is involved in methylation of homocysteine, a putative risk factor for cardiovascular disease, to methionine through a betaine-dependent pathway (one-carbon metabolism). It is unknown whether dietary intake of choline meets the recommended Adequate Intake (AI) proposed for choline (550 mg/day for men and 425 mg/day for women). The Estimated Average Requirement (EAR) remains to be established in population settings. Our objectives were to ascertain the reliability of choline and related nutrients (folate and methionine) intakes assessed with a brief food frequency questionnaire (FFQ) and to estimate dietary intake of choline and betaine in a bi-ethnic population.

**Methods:**

We estimated the FFQ dietary instrument reliability for the Atherosclerosis Risk in Communities (ARIC) study and the measurement error for choline and related nutrients from a stratified random sample of the ARIC study participants at the second visit, 1990–92 (N = 1,004). In ARIC, a population-based cohort of 15,792 men and women aged 45–64 years (1987–89) recruited at four locales in the U.S., diet was assessed in 15,706 baseline study participants using a version of the Willett 61-item FFQ, expanded to include some ethnic foods. Intraindividual variability for choline, folate and methionine were estimated using mixed models regression.

**Results:**

Measurement error was substantial for the nutrients considered. The reliability coefficients were 0.50 for choline (0.50 for choline plus betaine), 0.53 for folate, 0.48 for methionine and 0.43 for total energy intake. In the ARIC population, the median and the 75^th ^percentile of dietary choline intake were 284 mg/day and 367 mg/day, respectively. 94% of men and 89% of women had an intake of choline below that proposed as AI. African Americans had a lower dietary intake of choline in both genders.

**Conclusion:**

The three-year reliability of reported dietary intake was similar for choline and related nutrients, in the range as that published in the literature for other micronutrients. Using a brief FFQ to estimate intake, the majority of individuals in the ARIC cohort had an intake of choline below the values proposed as AI.

## Background

Population-level measurements of dietary intake of the essential nutrient choline and its metabolite, betaine, are of interest since a food composition database has only recently become available [1,2]. As a result, the habitual intake of these micronutrients and their health effects at the population level are not well established. In animal studies, low dietary intake of choline and betaine ostensibly results in aberrant DNA methylation and possible increased atherogenesis [3]. Independently of folate, dietary intake of choline and betaine are inversely associated with plasma homocysteine [4-6], a putative cardiovascular disease risk factor [7,8]. While it is true that it is unknown whether dietary intake of choline in the U.S. population meets the recommended Adequate Intake (AI) proposed for this nutrient, 550 mg/day for men and 425 mg/day for women [9], the studies conducted suggest that an important percent of the population may be choline deficient [10-12]. The Estimated Average Requirement (EAR), whose calculation requires a higher level of evidence, remains to be established in populations [13].

The reliability (reproducibility) of micronutrient intake, as assessed with a food frequency questionnaire, is lower compared with that of macronutrients [14-16]. Because the random effect (the error prone variance – covariance structure) of dietary assessment has been shown to have an important impact on risk estimates [17,18], several studies have estimated and have adjusted for measurement error in the assessment of dietary intake. The objective of our study was to ascertain the reliability of the dietary assessment for choline as assessed with a brief food frequency questionnaire (FFQ) and to estimate population dietary choline and betaine intakes. We also aimed to assess the FFQ measurement error and study the intraindividual variability when several related nutrients are considered simultaneously.

## Methods

Our study is based on data from the cohort component of the Atherosclerosis Risk in Communities (ARIC) Study. ARIC is an observational bi-racial cohort of 15,792 men and women between the ages of 45 and 64, sampled from four U.S. communities, Forsyth County, NC, Jackson, MS, suburbs of Minneapolis, MN, and Washington County, MD [19]. Enrollment reflected the demographics of the communities from which they were selected with the exception of the city of Jackson where enrollment was limited to black/African American residents. Baseline home interviews and examinations at specialized field centers occurred during 1987–1989; response rates were 46% of all eligible subjects in Jackson and approximately 66% in the remaining sites. The population included in these analyses is composed of 15,706 men and women aged 45–64 years with dietary data at the baseline visit.

The habitual dietary intake of choline and betaine was estimated and quantified with a 66-item food-frequency-questionnaire (FFQ), based on the Willett 61-item FFQ and expanded to include some ethnic foods. This dietary assessment instrument was applied for all the ARIC participants at baseline and on a random sample of 1,004 participants three years later (1990–1992). The participants were asked how often, on average, they had consumed listed items during the previous year. Nine frequency responses were listed ranging from more than six per day to almost never. We calculated daily servings by converting the consumption frequency to servings per day. Dietary choline and betaine were estimated as the sum of daily intakes using a choline and betaine database composed of the USDA choline and betaine content in common foods database (207 food items) and the University of Minnesota Nutrition Data System database for the portion sizes.

As part of the ARIC Dietary Assessment Repeatability Study, the mentioned random sample composed of 1,004 subjects (522 males and 482 females) was selected in equal number of participants from each ARIC locale, and studied three years after the baseline examination. The dietary form was administered in an identical manner as done during the ARIC baseline examination. The intraindividual variability (between-person and dietary instrument variability) was calculated and the reliability coefficient, the correlation between measures made at repeat visits, was estimated using mixed models regression [20].

For regressions models with several nutrients considered at one time, accounting for measurement error is complex. One needs to know not only the reliability for each independent variable in the model but also the measurement variation of the covariance between them, which is necessary in the measurement error estimation [21]. The total variance could be written as σ^2^_Total(T) _= σ_BP_^2 ^+ σ_e_^2 ^where σ_BP_^2 ^is the between-person component of variation and σ_e_^2 ^is the intraindividual component, sometimes called the measurement error. Using our repeatability study, a conditional reliability matrix was constructed; this matrix was derived from the measurement error estimates. To consider the joint intraindividual variation in several variables the total variance-covariance matrix of that set (vector) of variables can be specified as a sum of the between-person variance-covariance matrix (Σ_Total _= Σ_BP _+ Σ_e_). In our mixed model (Additional file 1) all four interrelated parameters, choline, total energy intake, folate and methionine, were the dependent variables, subject was used as a random effect variable, and study center and examination visit as fixed effect variables. Algorithms shown in Appendix were used to estimate the general variance-covariance matrix for the dietary compounds. From this the between-person (σ_B_^2^) and the error variances (σ_e_^2^) and error covariances between the nutrients, as well as the ratios of the between to the total (σ_T_^2^) variances were produced. In a first step the mixed model had an unstructured composition. We used the output estimates (as an average) as starting values in a new mixed model with the same variables and a structured covariance shown in Appendix. From this last mixed model we obtained the between-person and error variances and covariances as well as the ratios of between to total (σ_B_^2^/σ_T_^2^) variances and error to total (σ_e_^2^/σ_T_^2^) variances. Both the correlation coefficient between visits for choline and other nutrients, ρ_chol _= cov_visit_/var_chol _= σ_B_^2^/σ_T_^2^, as well as the total variance were calculated. Following the modeling to assess the joint intraindividual variability of the interrelated nutrients, a model was constructed with choline as the only dependent variable and technician nested within center, one of the main randomness factors in questionnaire-based dietary assessments, added to the random effect variables.

## Results

Of the 1,004 participants in the repeatability study, 482 (48%) were female and 294 (29%) were African Americans. The mean age was 55 years. Only for choline and methionine the mean and the standard deviation of energy-adjusted nutrient intakes were different between repeat visits Table 1.

**Table 1:** Comparison between the baseline visit and the second visit (mean and standard deviation) for the 1,004 ARIC participants in the repeatability study

**Variables**	**Baseline Visit**	**Second Visit**	**p-values^#^**
Total energy intake (kcal/d)	1,651 (593)	1,547 (576)	<0.0001
Choline intake (mg/d)^†^	319 (74)	270 (67)	<0.0001
Betaine intake (mg/d)^†^	112 (42)	108 (39)	0.16
Folate intake* (μg/d)^†^	230 (83)	223 (88)	0.13
Methionine intake* (mg/d)^†^	1,706 (441)	1,591 (439)	<0.0001
Vitamin B_6 _intake (mg/d)^†^	1.71 (0.50)	1.69 (0.69)	0.50

^#^p-values were calculated using a t-test

^†^Adjusted for total energy intake

Some variables (marked with *) have fewer observations

In mixed models used to assess the joint intraindividual variability for all interrelated nutrients Tables 2 we found a reliability coefficient of 0.50 for choline, 0.43 for caloric intake and 0.53 for folate (0.50 for total choline; choline plus betaine). These coefficients were similar to Pearson correlation coefficients estimated between the two visits (0.48 for choline and 0.49 for folate). The reliability coefficients for all studied nutrients showed similar values to those typically seen in the nutritional epidemiologic literature for micronutrients. The correlations between the observed values of the three micronutrients were 0.55, 0.91 and 0.53 for choline-folate, choline-methionine and folate-methionine, respectively. The measurement error variances had high values for all considered nutrients; 6,228 for choline, 145,397 for total energy intake, 5,504 for folate and 221,628 for methionine (Table 2). Somewhat surprisingly, the technician nested within center measurement error component was negligible, representing less than 0.01% of the total variance (results not shown).

**Table 2:** Components of reliability and measurement error expressed as ratios of between-person variance or covariance to total (co)variance for related dietary nutrients

	***Choline***	***Total Energy Intake***	***Folate***	***Methionine***
***Choline***	*6,228* ***0.50***	21,749	2,904	32,956
***Total Energy Intake***	0.44	*145,397* ***0.43***	15,079	131,704
***Folate***	0.48	0.47	*5,504* ***0.53***	15,587
***Methionine***	0.50	0.47	0.45	*221,628* ***0.48***

Note 1: The ratios of between person-variance to total variance are the reliability coefficients (presented in bold italic)

Note 2: The value presented in the upper-right half of the table are the values for the error variance (italic) and covariance error terms

In the full ARIC cohort the median and the 75^th ^percentile of dietary choline intake were 284 mg/day and 367 mg/day, respectively. In regression models, choline intake was associated with gender, race, study site, BMI, total energy intake, physical activity and, in women, with menopausal status (results not shown). Table 3 presents a series of statistics of interest for both choline and betaine. As expected, men had a higher intake compared to women. For men, African Americans had a lower intake for both choline and betaine. For comparison purposes, the percentages of participants below the AI were 94% of white men, 90% of white women, 93% of African American men, and 89% of African American women (results not shown).

**Table 3:** Distribution in the ARIC cohort, at baseline visit, of dietary choline and betaine by gender and race

	**Dietary Choline (mg/day)**			**Dietary Betaine (mg/day)**		
	median (IQR)	mean (SD)	75^th ^percentile	median (IQR)	mean (SD)	75^th ^percentile

*All ARIC participants* *(N = 15,706)*	284 (152)	304 (136)	367	94 (64)	106 (54)	132
						
*White Men* *(N = 5419)*	304 (158)	325 (140)	391	102 (70)	115 (59)	143
						
*White Women* *(N = 6043)*	273 (141)	288 (115)	350	90 (60)	99 (48)	125
						
*African American Men* *(N = 1618)*	295 (164)	320 (154)	381	99 (67)	109 (58)	136
						
*African American Women* *(N = 2626)*	263 (149)	287 (151)	344	88 (56)	99 (53)	120

Note 1: the proposed A.I. for choline is 425 mg/day for women and 550 mg/day for men

Note 2: IQR represent the interquartile range and SD the standard deviation

## Discussion

We assessed the reliability of a brief food frequency questionnaire and estimated the dietary intake of choline and betaine in a biracial middle aged cohort of men and women from four US locales. In our population-based cohort the majority of participants had an intake below the value proposed as the adequate intake (AI), used for comparison purposes. The reliability coefficients between visits were in the range of those for other micronutrients but lower compared with those found, for example, for laboratory analytes [22]. The measurement error variance values were substantial for all considered nutrients in concordance with previous studies that suggested that the FFQ is underestimating the actual dietary intakes. The variances of the mean dietary intakes of choline were relatively high in all race-gender groups, a finding that was expected due to the relatively modest reliability coefficients.

Choline, an essential nutrient for humans [13,23], is included in several compounds that belong to methyl-donors group. Betaine, a methyl-donor that is continuously produced from choline [24], has been shown to lead to immediate and long term lowering of plasma homocysteine after supplementation in the dietary intake range of betaine [5]. Through aberrant methylation of DNA, a low dietary intake of methyl-donors alters epigenetic regulation of a series of genes by which the atherogenic mechanism may be accelerated [25,26]. Folate and choline are metabolically interrelated [27]. When folate availability diminishes, there is an increased demand for choline as a methyl donor [28]. When choline availability is decreased, the demand for folate methyl-groups is increased [29]. Because methyl donation by folate and choline can be interchangeable, both folate and choline should be considered in epidemiological studies assessing the relationship between dietary intake of these compounds and cardiovascular disease (CVD). Accurate analysis of choline intake was previously not possible because the choline content of most foods was not known until recently [1,2]. During the past five years, several major population studies reported on the associations between high dietary intakes of choline and betaine and pathological conditions or markers of disease such as plasma total homocysteine (an inverse association in the Framingham Heart Study [11] and in the Nurses' Health Study [12]), incident coronary heart disease (marginally positive association in the Atherosclerosis Risk in Communities Study [30] and in the European Prospective Investigation into Cancer and Nutrition [31]), colorectal adenoma (positive association in the Nurses' Health Study [32]) and neural tube defects in offspring (negative association in a case-control study in California [10]). In a study that assessed the variability of dietary intake of choline in human subjects [33] in a clinical research setting, when healthy male and female volunteers were asked to select *ad libitum *a variety of foods, the standard deviations of choline in the total measured diet were 157 mg/day for males and 88 mg/day for females corresponding to a mean dietary intake of 631 mg/day for men, respectively 443 mg/day for women. These values are in the range of the AI for choline that has been tentatively set at 425 mg/day for women and 550 mg/day for men [13]. The EAR, one of the Dietary Reference Intake (DRI) categories for choline that requires a higher level of evidence for its calculation, remains to be established in population settings.

Much epidemiologic research is based on estimation of an association between a putative risk factor and a health outcome – for example, dietary intake of a certain nutrient and coronary heart disease. Regression techniques, including Cox regression, produce biased estimates of exposure-disease relationships when the exposure variable has a high variability, which is equivalent with a low repeatability [21]. The reliability coefficients were relatively low in our study, in the range 0.43–0.53. As a consequence regression calibration procedures, using these coefficients, should be used to adjust for the measurement error [34-36]. The algorithm we used permitted the partition of the total variance into the between-person component and the error component. For each of the nutrients considered these two components were of comparable magnitude, implying a relatively large measurement error.

As expected, the dietary choline intake was associated with factors such as gender, menopausal status, total energy intake and BMI. These associations might be explained by the consequence of a direct proportionality between the total quantity of ingested foods and the amount of choline within it than by the capacity of premenopausal women to internally synthesize choline moieties which have an impact on the choline plasma levels.

Several limitations of our study should be acknowledged. The interval between dietary assessments in the ARIC study was long, which may have resulted in changes in dietary intake over time. This may have contributed to the modest level of repeatability we observed. Another limitation is the use of a food frequency questionnaire to estimate intakes of choline and betaine in general. Not all foods containing choline and betaine are assessed with the ARIC FFQ. However, foods that were high in choline such as liver and eggs and would contribute significantly to the population intake were included. The validity of this questionnaire to assess intake of choline and betaine is unknown and remains of interest for future studies.

There are several strengths to our investigation. Prior to this study, information about the repeatability of the short version of the Willett FFQ as it pertains to dietary choline and betaine intake was lacking. There is also novelty in estimating intraindividual variability and correcting for measurement error bias as it pertains to choline and betaine. We report both the correlations between the two visits as well as the magnitude of error (variance components) in the dietary assessment which have an application for future studies.

## Conclusion

For choline and for choline plus betaine the relative low reliability was similar to those of folate and methionine, and in the range of those reported for other micronutrients. In the estimation of these nutrients, adjustment for measurement variability (using, for example, a calibration method) should be used whenever possible. The majority of the ARIC participants were below the AI: 93% of white men, 88% of white women, 92% of African American men, and 87% of African American women.

## Competing interests

Dr. S. H. Zeisel received grant support from Mead Johnson Nutritionals and the Egg Nutrition Research Center for studies other than those described in this paper. The other authors declare that they have no competing interests.

## Authors' contributions

AB, LEC and GH conceived of and designed the study. AB and LEC performed the statistical analyses. AB, LEC, AMSR, SHZ and GH interpreted the results. AB drafted the manuscript. All authors revised the manuscript for intellectual content, and read and approved the final manuscript.

## Supplementary Material

Additional File 1 **Measurement error model and variance-covariance matrix.** The description provided represents the algorithms used to construct the measurement error mixed model and to obtain the variance-covariance matrix for the related dietary nutrients. Click here for file
