# Supplementary material for: Repeatability and measurement error in the assessment of choline and betaine dietary intake: the Atherosclerosis Risk in Communities (ARIC) Study
Source: Nutr J. 2009 Feb 20;8:14. doi: 10.1186/1475-2891-8-14 (PMC2654540; doi:10.1186/1475-2891-8-14)
Supplement: Additional File 1 — Measurement error model and variance-covariance matrix. The description provided represents the algorithms used to construct the measurement error mixed model and to obtain the variance-covariance matrix for the related dietary nutrients. [file 1475-2891-8-14-S1.doc]

**Appendix**

When one variable is considered, the measurement error model is:

Y_ij_ = y_i_ + ε_ij_ = µ + α_i_ + ε_ij_ , Observed = True + Error,

N(μ, σ^2^_BP_) N(0, σ^2^_e_)

Where variance Y_ij_ ( = σ^2^_Total_) = σ^2^_BP_ + σ^2^_e_ , i = person, j = measurement on person.

The multivariate measurement error model could be written as:

Y_1ij_ y_1i_  ε_1ij_

. . .

. = . + .

. . .

Y_kij_  y_ki_  ε_kij_

N(μ, Σ_BP_) N(0, Σ_e_)

Σ_Total_ = Σ_BP_ + Σ_e_

When one variable is considered the measurement error model for choline could be slightly expanded, with a a center effect and also systematic visit differences:

Choline_ijl_ = µ + α_i_ + ß Visit_ij_ + γ_l_ Center_il_ + ε_ij_ , (1)

where Choline_ijl_ is the dietary intake of choline, i = person, j = visit and l = center (from the repeatability matrix). The person random effect was calculated using a population with a normal distribution with mean zero and variance σ_B_^2^, and the visit effect will be calculated assuming a population normally distributed with mean zero and within person variance σ_e_^2^. The variance and the covariances are obtained from the covariance matrix of equation 1:

Var (Choline_ij_) = Var (α_i_ + ε) = Var (α_i_) + Var (ε) = σ_B_^2^ + σ_e_^2^ = σ_T_^2^,

Cov (Choline_i1_, Choline_i2_) = Cov (α_i_ + ε_i1_, α_i_ + ε_i2_) = Cov (α_i_, α_i_) + Cov (α_i_, ε_i1_) + Cov (α_i_, ε_i2_) + Cov (ε_i1_, ε_i2_) = Var (α_i_) = σ_B_^2^ = between person variance,

assuming α_i_ and ε independent, as well as ε_i1_ and ε_i2_ independent. The reliability coefficient, ρ, is: Corr (Choline_i1_, Choline_i2_) = Cov (Choline_i1_, Choline_i2_) / Var (Choline_ij_) = σ_B_^2^ / (σ_B_^2^ + σ_e_^2^). This could be obtained with SAS Proc Mixed, where σ_B_^2^ is CS (ID) and the σ_e_^2^ represents RESIDUAL.

We considered our sample from two visits from ARIC as a normally distributed population with the following mean and variance:

choline_i1_ σ_1_^2^ σ_12_

~ N mean,

choline_i2_ σ_12_ σ_2_^2^

where i^th^ subject, and 1 = visit 1, 2 = visit 2. The term σ_12_ represents the between visits covariance and σ^2^ the total variance (between-person plus error).

When another nutrient or nutrient-related variable (for example, calories) is considered in the vector, and the visit and center term included:

Chol_ij_ μ_ch_+α_ch_+ß_ch_Visit+Σγ_ch_Center_ch_+ε_chij_

=

Cal_ij_ μ_cal_ + α_cal_ + ß_cal_Visit + Σγ_cal_Center_cal_ + ε_calij_

where chol = choline, cal = calories.

Therefore:

α_ch,i_ 0 σ_B_,_ch_^2^ σ_B,ch,cal_

~ N ,

α_cal,i_ 0 σ_B,ch,cal_ σ_B,cal_^2^

ε_ch,i_ 0 σ_e,ch_^2^ σ_e,ch,cal_

~ N ,

ε_cal,i_ 0 σ_e,ch,cal_ σ_e,cal_^2^

With two nutrients and two measurements, we have:

chol_i1_ σ_ch_^2^ ρ_ch_σ_ch_^2^

Var chol_i2_ = ρ_ch_σ_ch_^2^ σ_ch_^2^

cal_i1_ σ_chcal_ σ_chcalB_ σ_cal_^2^

cal_i2_ σ_chcalB_ σ_chcal_ ρ_cal_σ_cal_^2^ σ_cal_^2^

where σ_ch_^2^ = σ_B_,_ch_^2^ + σ_e_,_ch_^2^ and σ_cal_^2^ = σ_B_,_cal_^2^ + σ_e_,_cal_^2^;

where Cov (Chol_i1_, Chol_i2_) = ρ_ch_ σ_ch_^2^ and Cov (Chol_ij_, Cal_ij_) = σ_chcal_ = σ_chcalB_ + σ_chcale_;

and where Cov (Chol_i1_, Cal_i2_) = σ_chcalB_ .
